# Supplementary material for: Correlation between DNA Methylation and Cell Proliferation Identifies New Candidate Predictive Markers in Meningioma
Source: Cancers (Basel). 2022 Dec 17;14(24):6227. doi: 10.3390/cancers14246227 (PMC9776514; doi:10.3390/cancers14246227)

Supplementary Figure S1. Principal component analysis of the top 2099 CpGs from the differential analysis between grade 1 and grade 2+3 meningiomas.

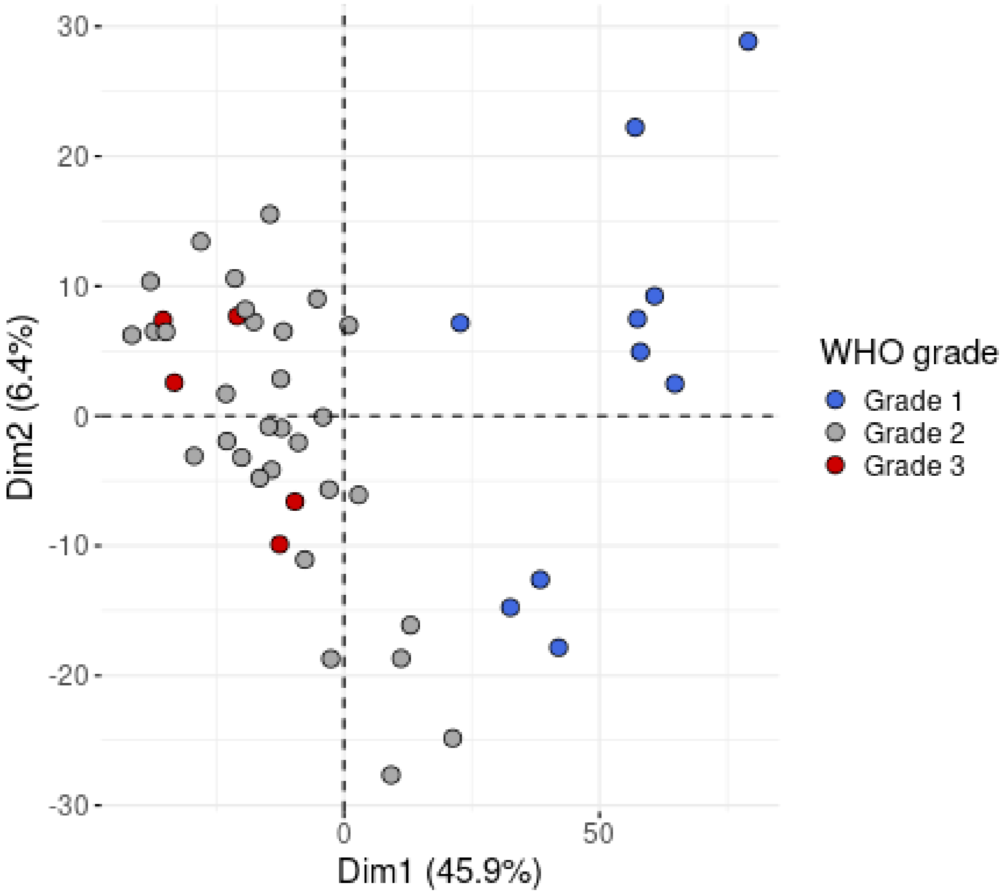

Supplement: Supplementary file 1 [file cancers-14-06227-s001.zip › Supplementary Figure S1.pdf]
