# Supplementary material for: Correlation between DNA Methylation and Cell Proliferation Identifies New Candidate Predictive Markers in Meningioma
Source: Cancers (Basel). 2022 Dec 17;14(24):6227. doi: 10.3390/cancers14246227 (PMC9776514; doi:10.3390/cancers14246227)

Supplementary Figure S2. Validation of the proliferative signature on an external methylation dataset (https://www.ncbi.nlm.nih.gov/geo/query/acc.cgi?acc=GSE200321; from Daoud et al. [30]). Hierachical clustering against the 310 CpGs and 60 samples of various meningioma grades and histological types (6 grade 1: 5 meningothelial and 1 transitional; 50 grade 2: 10 chordoid, 27 atypical and 13 atypical and invasive; 4 grade 3: anaplastic). Raw data from this dataset was processed with the same analytical pipeline as the main dataset presented in this work.

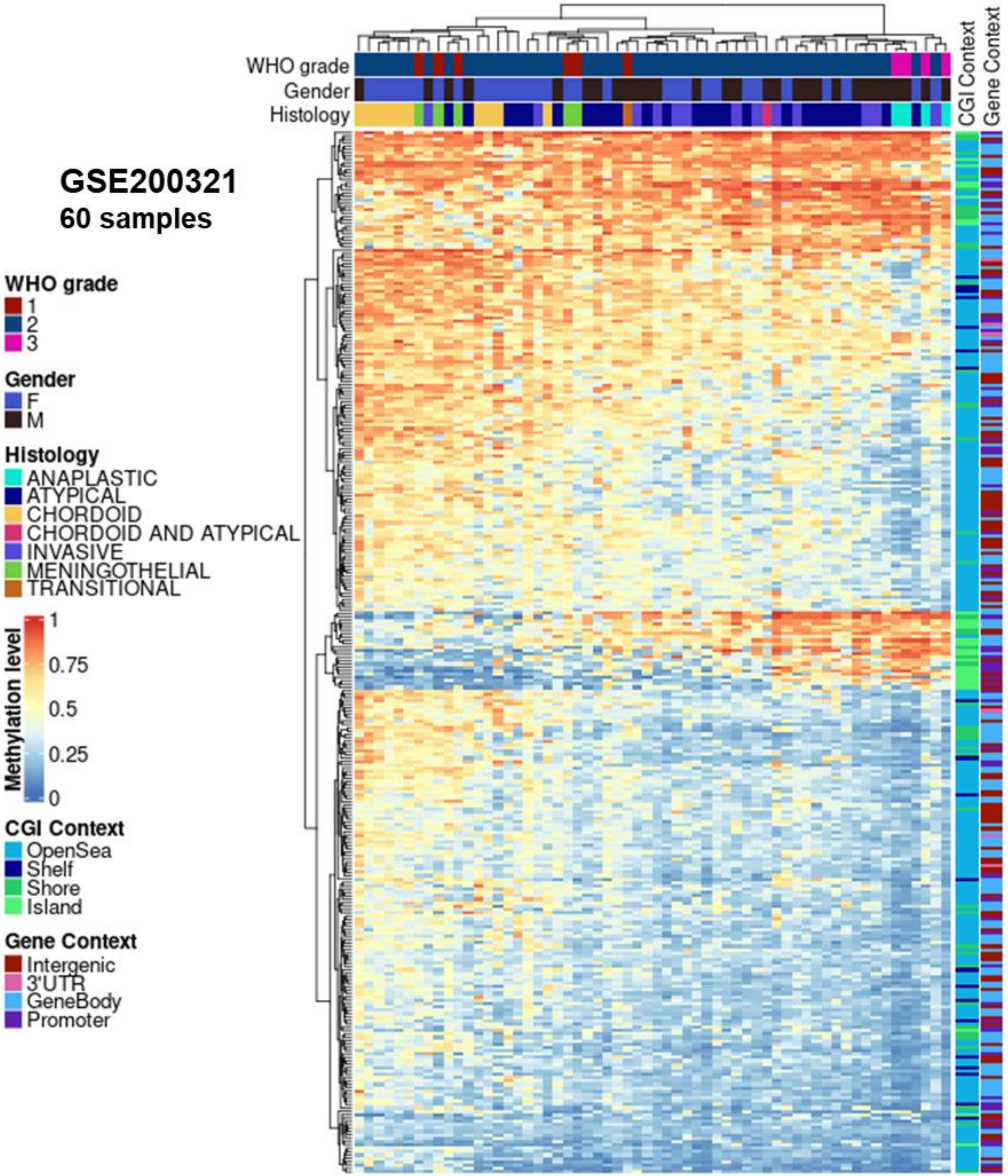

Supplement: Supplementary file 1 [file cancers-14-06227-s001.zip › Supplementary Figure S2.pdf]
